# Deletion of myeloid-PTP1B decreases MHC Class I expression and peptide presentation through an IL-10 dependent mechanism in response to LPS challenge

**DOI:** 10.1186/1476-9255-12-S1-P1

**Published:** 2015-04-16

**Authors:** Samantha Le Sommer, Cristina Martin-Granados, Mirela Delibegovic

**Affiliations:** 1 Institute of Medical Sciences (IMS) School of Medical Sciences, University of Aberdeen, UK

## 

Protein Tyrosine Phosphatase 1B (PTP1B) inhibition is a target in the treatment of type 2 Diabetes Mellitus, and as such PTP1B inhibitors are in Phase II clinical trials. Previously our laboratory demonstrated that myeloid-specific deletion of PTP1B (LysM PTP1B) results in an increase in systemic IL-10 secretion and expression. In the current work we investigated how PTP1B deficiency affects the activation phenotype of murine macrophages in response to inflammatory stimuli. We demonstrate that myeloid-specific PTP1B deletion results in a decrease in expression of MHC Class I, along with co-stimulatory molecules CD80 and CD40. Interaction assays reveal a defect in the cells’ ability to activate reporter B3Z T cells. Myeloid-specific PTP1B deletion increases the percentage of bone-marrow-derived-macrophages (BMDMs) positive for IL-10 which is associated with a decrease in iNOS production. Western blotting analysis demonstrated hyperphosporylation of ERK1/2 which has been suggested before to improve access to the IL-10 promoter. This provides evidence to suggest that myeloid-PTP1B deletion decreases MHC Class I expression and peptide presentation through an IL-10-dependent mechanism.

